# Fertilizer types and nitrogen rates integrated strategy for achieving sustainable quinoa yield and dynamic soil nutrient-water distribution at high altitude

**DOI:** 10.1038/s41598-025-89572-2

**Published:** 2025-02-15

**Authors:** Xiaojing Sun, Chenglei Deng, Jiaxing Gao, Jingying Lu, Yan Zheng, Zeyun Guo, Yadi Sun, Chuangyun Wang, Yan Deng

**Affiliations:** College of Agriculture, Shanxi Agricultural University, Taiyuan, 030031 China

**Keywords:** Quinoa, Bio-microbial fertilizer, Slow-release fertilizer, Soil enzyme activity, Soil nutrients, Water use efficiency, Quinoa yield, Physiology, Environmental sciences

## Abstract

Quinoa (*Chenopodium quinoa* Willd.) is a crop particularly adapted to high-altitude environments characterized by significant variability in climate and soil conditions Fertilization is essential for providing nutrients and influencing soil nutrient cycling and hydrological dynamics. This study aimed to optimize fertilizer type and nitrogen (N) application rates to improve soil nutrient availability, moisture retention, and quinoa yield. We examined three fertilizer types: compound fertilizer (NPK), bio-microbial fertilizer (BM), and slow-release fertilizer (SRF), with nitrogen application rates of 90, 120, and 150 kg ha^− 1^, compared to a control group (CK) with no fertilization. Our results revealed that applying 120 kg ha^− 1^ of nitrogen with SRF significantly reduced soil bulk density, improved water retention beyond 60 cm depth, and enhanced water use efficiency by 9.2–16.2%, alleviating water stress. In conjunction with BM, this nitrogen application increased soil organic matter, alkali-hydrolyzed nitrogen, and the availability of phosphorus and potassium, especially during the grain-filling stage, promoting quinoa growth. Elevated nitrogen rates (120 and 150 kg ha^− 1^) with BM maximized soil urease and sucrase activities, correlating positively with key soil chemical parameters. Additionally, 120 kg ha^− 1^ of SRF notably boosted quinoa biomass and yield components. Economic analysis indicated that SRF at 120 kg ha^− 1^ nitrogen provided the highest productivity. These results highlight the importance of fertilizer type and nitrogen rates in enhancing soil nutrient status and optimizing water infiltration in high-altitude soils, offering a drought-resistant strategy for quinoa cultivation.

**Supplementary Information:**

The online version contains supplementary material available at 10.1038/s41598-025-89572-2.

## Introduction

Quinoa (*Chenopodium quinoa* Willd.) is a highly resilient plant with unique ecological adaptability^1,2^. It can thrive in some of the most extreme and harsh weather conditions, including significant diurnal temperature variations, high salinity, and frost^3^. As a result, quinoa has emerged as an excellent candidate for cultivation in arid agricultural regions^4^. While it is well-known for its suitability in high-altitude areas, quinoa is not restricted to the Andes Mountain range; it also has the potential to flourish in other mountainous regions of developing countries. China began trial planting of quinoa in the 1980s, recognizing its unique nutritional value and potential as a new crop. Since 2008, large-scale cultivation efforts have been implemented in Shanxi Province, leading to significant increases in both the area planted and crop yield^5^. However, in these high-elevation regions, farmers face challenges such as water scarcity, low rainfall, and limited access to artificial irrigation^6^. Drought is a critical stress factor that severely affects crop growth and yield at high altitudes, while insufficient precipitation raises the risks associated with planting and introduces uncertainty in the yields of dryland crops^7^. Therefore, in high-altitude areas with low water use efficiency, how to improve water use efficiency and maintain soil and crop productivity is the biggest challenge facing quinoa growth.

Many studies on crops have reported that drought can cause irreversible effects, such as decreased leaf morphology, reduced leaf number, and an increased root-to-shoot ratio^8^, these effects vary with soil moisture and crop growth stage, as numerous interactions occur between water and nutrients^9^. The prediction was that quinoa exhibited robust tolerance to water stress during the heading and flowering stages, whereas drought stress encountered at the branching and ripening stages would notably diminish the crop yield^10^. Certain researchers highlight numerous avenues for enhancing quinoa production or minimizing water consumption^11^. Over the past few decades, there has been a substantial increase in the irrigation efficiency and water productivity of quinoa systems in Asia and Australia, attributed to advancements in varieties and refined management practices related to irrigation, nutrient application, and other factors^10^. However, the adjustment of water distribution patterns through the utilization of different fertilizer types to enhance water use efficiency is an exceptionally uncommon practice in the field of quinoa research. The application of various fertilizers and alterations in water content significantly influence soil water and nutrient movement and distribution within the crop system^12^. Conventional urea, particularly, undergoes rapid decomposition upon exposure to precipitation, posing a threat to both soil and groundwater environments^13^. When urea applied as a base fertilizer, urea can lead to excessive nitrogen (N) levels during early crop growth, increasing the risk of nitrate leaching^14^. Slow-release fertilizers (SRF) hinder water transport necessary for urea dissolution within their coating, thereby extending the duration of N fertilizer effectiveness^15^. The SRF continuously and stably releases nutrients throughout the entire growth period of plants, promoting the absorption and utilization of water^16^. Bio-microbial fertilizers (BM) affect crop water use efficiency by influencing crop yield^17^, BM can significantly increase soil organic matter (SOM) and other nutrient contents, which in turn enhances the absorption and utilization of alkali hydrolyzed N (AH-N) and available potassium (AK) by both plants and soil^18^. Therefore, the application rate of N fertilizer and the choice of fertilizer are crucial for soil moisture and nutrient management. However, the impact of different fertilizers on the dynamic distribution of soil moisture and nutrients at various N application levels remains unclear.

The content of SOM, AH-N, available phosphorus (AP), and AK are the main nutrients in soil^19^. Soil nutrient balance is crucial in determining the direction of soil fertility evolution and is closely intertwined with the benefits derived from fertilization, as well as ecological and environmental safety^20^. Traditionally, the focus of fertilization has primarily been on enhancing crop yields, which has, to some extent, overshadowed concerns related to maintaining soil fertility balance^21^. Soil enzymes are also effective indicators for evaluating soil fertility and plant growth. They serve as catalysts for soil biochemical reactions and directly reflect soil metabolic needs and nutrient availability^22^. As indicators of process diversity, soil enzymes provide crucial information to the soil system regarding its biochemical potential and regulatory capacity^23^. Soil urease (S-UE) is a key hydrolytic enzyme in soil ecosystems, primarily promoting N transport within plant systems and serving as a major biocatalyst for the decomposition, turnover, and mineralization of soil organic matter^24^, soil sucrase (S-SC) can promote the hydrolysis of sucrose molecules into low molecular weight glucose and fructose, which can be absorbed and utilized by plants and microorganisms^25^. These soil enzyme activities are responsive to soil nutrients, fertility levels, and environmental changes. N fertilizer management is an important factor affecting soil microbial populations and enzyme activities^26^. Further research is warranted to explore the impact of various fertilizer types and nitrogen (N) application levels on soil nutrient dynamics and enzyme activity in high-altitude regions throughout different growth stages. Specifically, this study aims to examine the effects of distinct fertilizer formulations—namely NPK, biological manure (BM), and slow-release fertilizers (SRF)—in conjunction with varying N application rates (90 kg ha^−1^, 120 kg ha^− 1^, and 150 kg ha^− 1^) on soil moisture retention, nutrient availability, crop yield, and associated economic benefits. The objective is to enhance the infiltration of limited rainfall into deeper soil strata, improve water use efficiency, and promote sustainable soil quality and crop productivity. We hypothesize that the type of fertilizer applied can influence the dynamic distribution of soil moisture and facilitate the infiltration of rainfall into deeper layers. It is anticipated that soil moisture, nutrient levels, and enzyme activity associated with quinoa cultivation will vary significantly depending on the fertilizer type employed. Furthermore, even when N levels are standardized across different fertilizers, the specific type may exert a differential influence on these parameters, with potential variations corresponding to changes in N application levels. To test this hypothesis, we conducted a two-year field experiment with three primary objectives: (1) To analyze water distribution across various soil depth intervals (0–20 cm, 20–40 cm, 40–60 cm, 60–80 cm, and 80–100 cm) and assess the responsiveness of different fertilizer types to fluctuations in water content at varying N application levels; (2) To assess the variations in soil nutrient profiles and enzyme activity at various growth stages in relation to the differing fertilizer types and N application levels; and (3) To identify the optimal combination of fertilizer type and N application rate that maximizes water use efficiency, quinoa yield, and economic viability.

## Materials and methods

### Site description

**Fig. 1:**
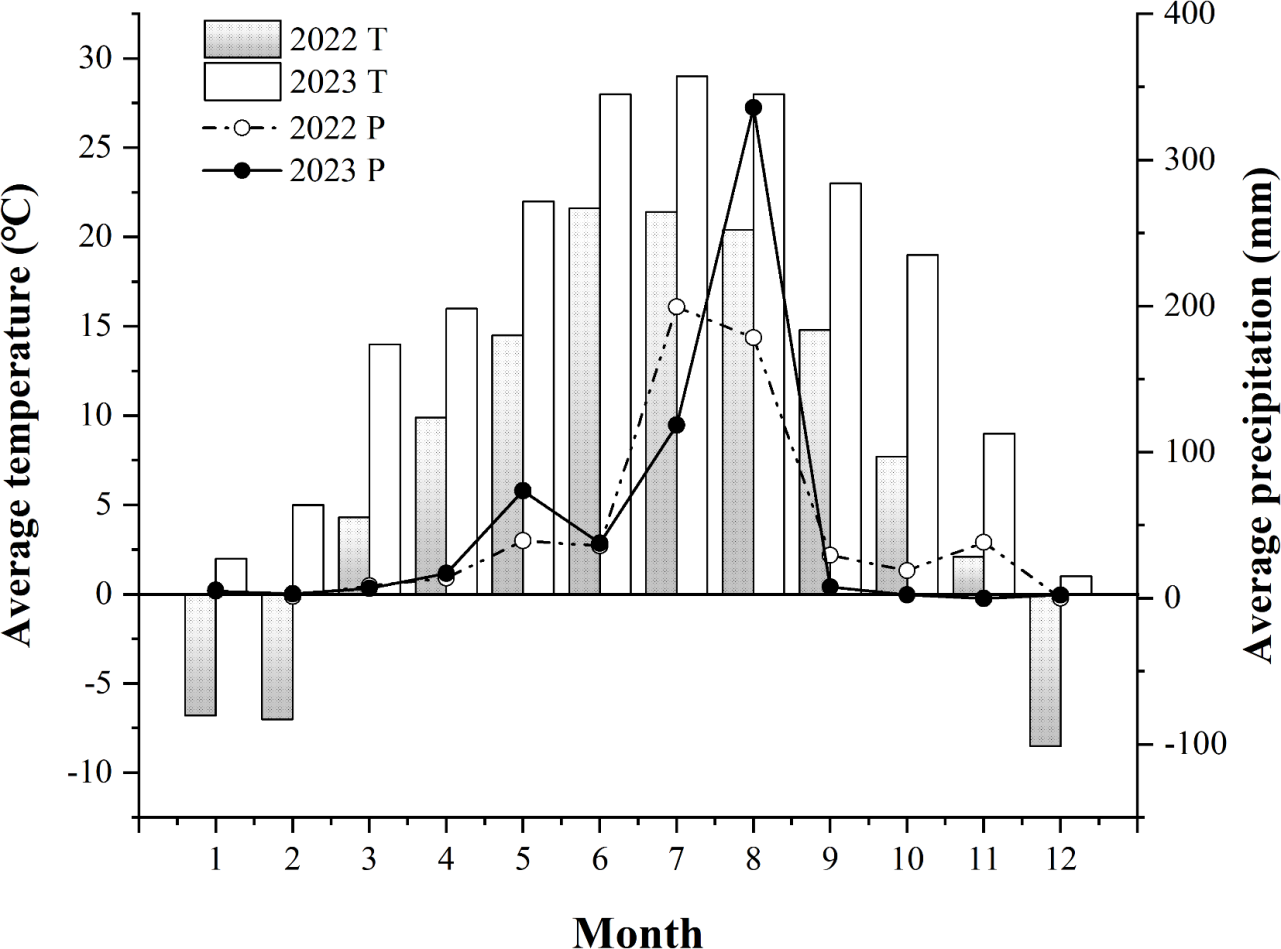
Average temperature (T) and average precipitation (P) in Jingle County, Shanxi Province in 2022 and 2023. The average means monthly average.

The experiment was conducted between 2022 and 2023 at an experimental facility located in Jingle County, Xinzhou City, Shanxi Province, China (38°3′N, 111°9′E). This facility is situated at an altitude of over 2000 m and experiences a temperate monsoon climate, characterized by four distinct seasons. Summers are warm to hot, with significant diurnal temperature variations. The region enjoys more than 2500 h of sunshine annually and has an average frost-free period of 120 to 135 days. Rainfall measured 569.5 millimeters in 2022 and 610.2 millimeters in 2023. The average annual temperatures for these years were 7.81 °C and 16.3 °C, respectively (Fig. 1). In 2022, basic nutrient measurements were taken from the 0–20 cm soil layer, revealing 7.60 g kg^− 1^ of organic matter, 91.5 g kg^− 1^ of total nitrogen, 128 mg kg^− 1^ of available potassium, 20.23 mg kg^− 1^ of available phosphorus, and a pH value of 8.14. In 2023, similar measurements were conducted in the same soil layer, which showed an organic matter content of 7.87 g kg^− 1^, a total nitrogen content of 95.5 g kg^− 1^, an available potassium content of 132 mg kg^− 1^, an available phosphorus content of 21.31 mg kg^− 1^, and a pH level of 8.09.

### Experimental design

The seeds used in this experiment are the quinoa variety (*BL*77) provided by Shanxi Huaqing Quinoa Product Development Co., Ltd. in China. Quinoa was sown in a flat field in June (2022–2023) after soil preparation and harvested in October each year. All plots were 50m^2^ (5 m×10 m), with a density of 6,9000 plants per hectare, sowing depth is 1–2 cm, a row spacing of 40 cm, and a plant spacing of 40 cm, a 1 m corridor left between each plot. The experiment involved two factors (three types of fertilizer × three levels of N application). The N application levels were as follows: N1 (90 kg ha^− 1^), N2 (120 kg ha^− 1^), and N3 (150 kg ha^− 1^), three fertilizer types were applied among the three N application levels, namely compound fertilizer (NPK, *N* ≥ 25%), bio-microbial fertilizer (BM, *N* ≥ 13%), and slow-release fertilizer (SRF, *N* ≥ 17%) (Table 1). which includes a total of 10 treatments, one of which is a non-fertilized control (CK). The experiment was conducted using a completely randomized design, with each treatment replicated three times. Throughout the entire growth period, no artificial irrigation was applied, and natural precipitation served as the sole source of water. Insecticides were utilized to manage pests and diseases, while weeds were controlled manually. All other cultivation and management practices adhered to local standards and practices.

**Table 1:** Design for the fertilizer and nitrogen managements.

Treatment		Fertilizer application	Nutrient content (kg ha^− 1^)		
Nitrogen level	Fertilizer type		*N*	*P*_2_O_5_	K_2_O
N0	CK	0	–	–	–
N1 (90 kg ha^− 1^)	NPK	360	90	36	57.6
	BM	692.31	90	34.62	48.46
	SRF	529.41	90	52.94	95.29
N2 (120 kg ha^− 1^)	NPK	480	120	48	76.8
	BM	923.07	120	46.15	64.61
	SRF	705.88	120	70.58	127.05
N3 (150 kg ha^− 1^)	NPK	600	150	60	96
	BM	1153.85	150	57.69	80.77
	SRF	882.35	150	88.24	158.83

### Agronomic traits and yield composition measurements

During the mature stage, select ten plants with consistent growth from each plot. Plant height and main spike length were measured using a tape measure. The number of branches was counted, starting from the bottom-most branch with spikes. To determine the leaf area, the fourth-to-last functional leaf of each quinoa plant was selected, and its area was measured using the CID Bio Science handheld laser leaf area analyzer CI-203, which imported from United States.

At harvest time, ten plants with consistent growth were randomly selected from each plot and enclosed in mesh bags. After drying the plants in sunlight, the quinoa ears were threshed and stored. Subsequently, the ears were weighed using a JM-a 20,002 electronic balance, and evaluated yield and thousand-grain weight based on seed samples. After withering the plants in an oven at 75 °C and drying them at 105 °C, the dried samples were weighed and the above-ground biomass was measured.

### Soil water content, soil bulk density measurement and calculation

Soil water content (SWC) was collected using a soil drill, with three repetitions per plot at depths ranging from 0 to 100 cm. Every 20 cm, a soil layer was extracted, packed into an aluminum box, weighed, and then dried in a 105 °C oven until it reached a constant weight, the SWC was then calculated based on these measurements. The sampling times were heading, anthesis, Pre-grouting, Post- grouting and maturity stage.

Soil bulk density (SBD) was measured using a ring cutter both before sowing in 2022 and after harvesting in 2023, with three replicates per plot. Each soil sample was dried to a constant weight at 105 °C, and subsequently, the SBD was calculated based on these measurements.

1 $$\:SBD=\frac{M}{V}$$

Among them, SBD is the soil bulk density (g cm^− 3^); M is the mass of the dried soil sample (g); V is the volume of the ring cutter (cm^3^).

### Soil nutrients, soil enzyme activity measurement and calculation

Soil nutrients are obtained from soil samples taken based on soil moisture content, all topsoil samples were air-dried and then sieved through a 0.15 mm sieve. The content of soil organic matter (SOM) was determined by the potassium dichromate volumetric method, while the soil’s alkaline N content (AH-N) was measured using the alkaline diffusion method. Additionally, the available phosphorus content (AP) in the soil was assessed via the molybdenum antimony colorimetric method, and the available potassium content (AK) was determined using the flame photometer method. All methods refer to the *Guidance of Plant Physioiogy Experiments*^27^.

Soil enzyme activity was measured in the 0–20 cm surface soil during the maturity period in 2023. Soil urease (S-UE) and sucrase (S-SC) were determined using spectrophotometry, using a Mettler ML204 10,000 balance and a Thermo Fisher Multiskan GO 1510 full microplate reader. All methods refer to the *Principles and Methods of Soil Microbial Research*^28^.

### Calculation of WUE

Soil water consumption (ET) and water use efficiency (WUE) are calculated using the following formula^29^:

2 $$\:W=SBD\times\:SD\times\:SWC$$

3 $$ET= \Delta W+P$$

4 $$\:\text{W}\text{U}\text{E}=\frac{\text{Y}}{\text{E}\text{T}}$$

Among them, W is the soil water storage capacity (mm); SBD is soil bulk density (g cm^− 3^); SD is the depth of the soil layer (cm); SWC is the soil moisture content (%); P is the precipitation during the growing season of quinoa; Y represents the grain yield. Considering the terrain of the experimental site, factors such as surface runoff and soil moisture infiltration are considered negligible.

### Statistical analysis

The data was organized using Microsoft Excel 2019, developed by Microsoft Corporation in the United States. For data processing, we utilized DPS 7.05, a statistical software developed in China, with significance set at *P* < 0.05. We then employed IBM SPSS Statistics 27, released by IBM in the United States, to simulate the data using a one-way general linear model. This analysis allowed us to construct factors, covariates, and their interactions to illustrate significant relationships between each factor and its interactions with various indicators. Finally, we used Origin 2021, developed by OriginLab Corporation in the United States, to create visualizations of the results.

## Results

### Soil bulk density (SBD)

**Fig. 2:**
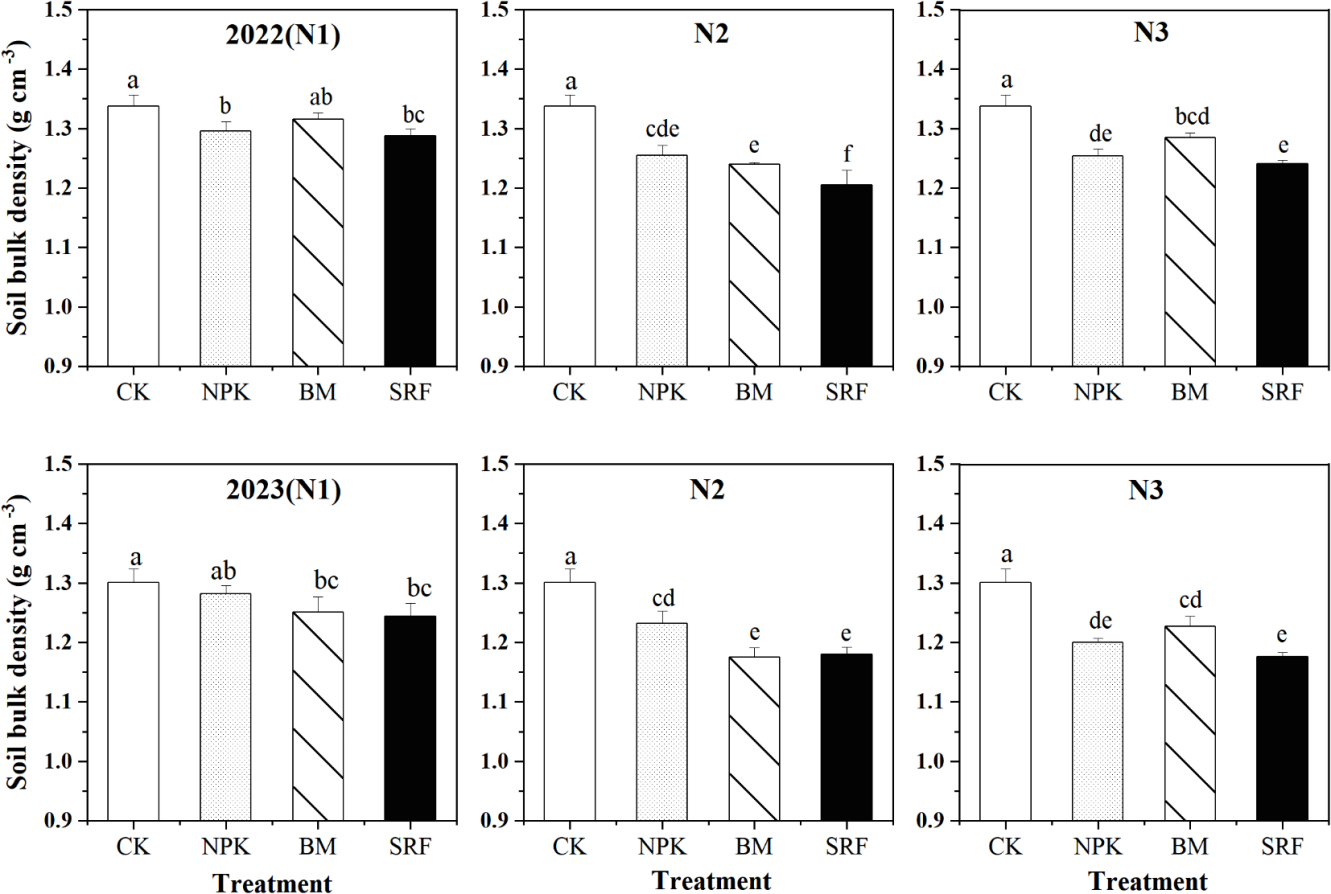
Effects of different nitrogen application levels and fertilizer types on Soil bulk density (SBD) of quinoa. The error bars for each treatment represent the standard error of the mean (*n* = 3). Different lowercase letters indicate significant differences between different treatments (*P* < 0.05).

The trend in soil bulk density (SBD) over the two growing seasons is consistent (Fig. 2). In 2022, the use of slow-release fertilizer (SRF) significantly decreased soil SBD compared to both compound fertilizer (NPK) and bio-microbial fertilizer (BM), with the N2 level proving most beneficial for topsoil structure when using SRF. In 2023, at the N1 level, the type of fertilizer had no significant effect on soil SBD. However, at the N2 level, both BM and SRF significantly reduced SBD, and there was no difference in SBD reduction between the SRF treatments at the N2 and N3 levels.

### Soil water content (SWC)

#### Heading stage

**Fig. 3:**
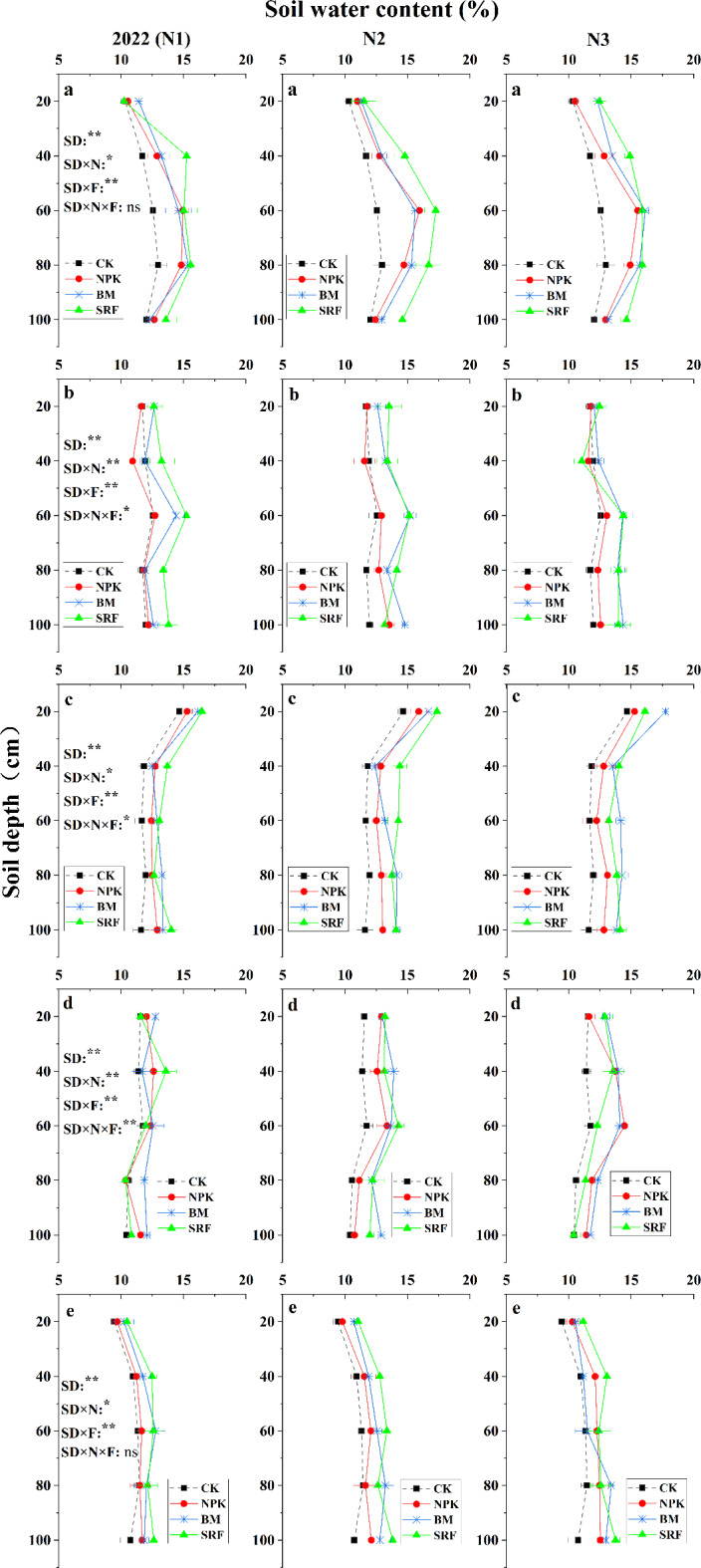
The effects of different nitrogen application levels and fertilizer types on the vertical distribution characteristics of soil moisture content in quinoa soil were investigated at various growth stages in 2022. The error bars associated with each process represent the standard error of the mean (*n* = 3). Heading stage (**A**), Anthesis stage (**B**), Pre-grouting stage (**C**), Post-grouting stage (**D**), Maturity stage (**E**). In the three-factor analysis of variance, SD, SD × N, SD × F, and SD × N × F represent the effects of soil depth, soil depth × nitrogen level, soil depth × fertilizer type, and the interaction between soil depth, nitrogen level, and fertilizer type on soil moisture content, respectively. * indicate significance at the 0.05 level, ** indicate significance at the 0.01 level, and ‘ns’ indicates non-significance.

**Fig. 4:**
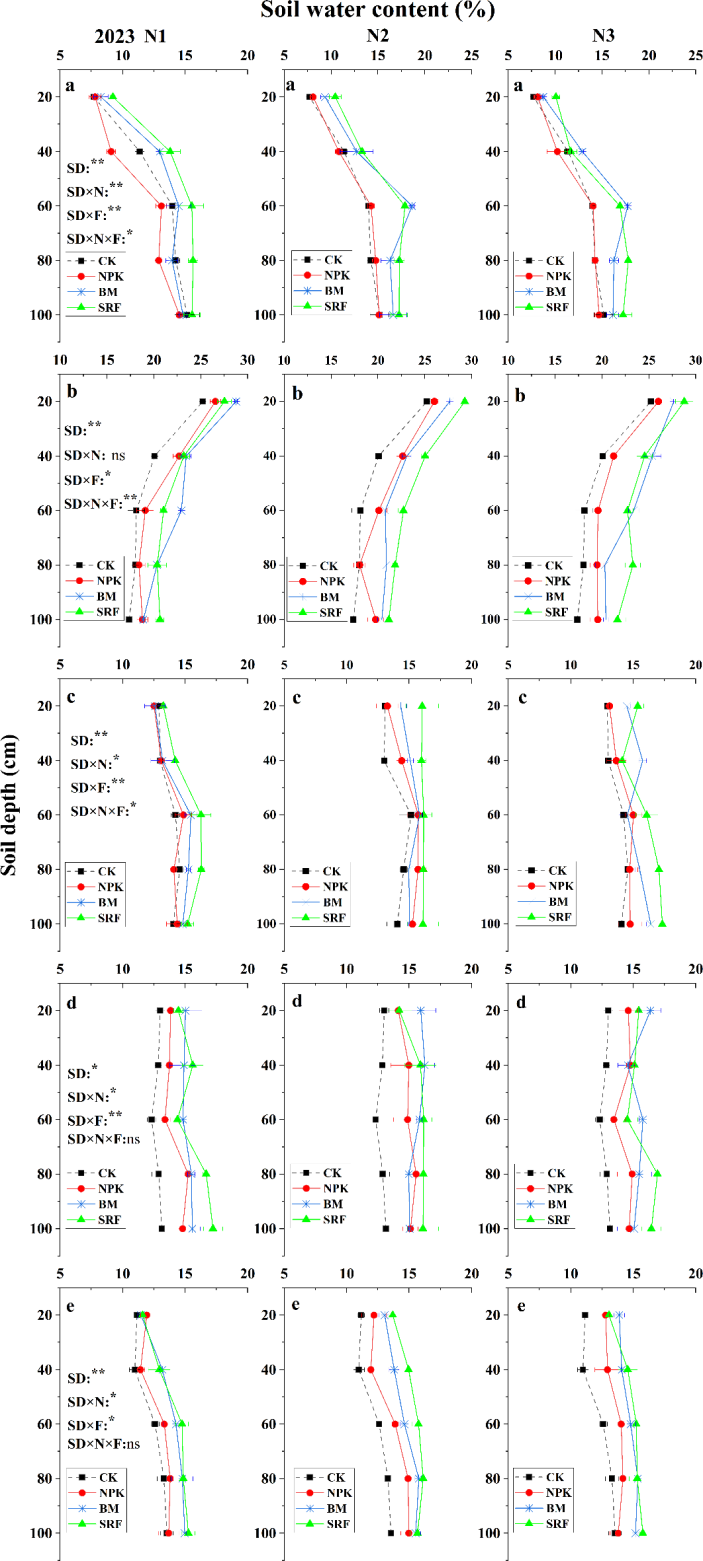
The effects of different nitrogen application levels and fertilizer types on the vertical distribution characteristics of soil moisture content in quinoa soil were investigated at various growth stages in 2023. The error bars associated with each process represent the standard error of the mean (*n* = 3). Heading stage (**A**), Anthesis stage (**B**), Pre-grouting stage (**C**), Post-grouting stage (**D**), Maturity stage (**E**). In the three-factor analysis of variance, SD, SD × N, SD × F, and SD × N × F represent the effects of soil depth, soil depth × nitrogen level, soil depth × fertilizer type, and the interaction between soil depth, nitrogen level, and fertilizer type on soil moisture content, respectively. * indicate significance at the 0.05 level, ** indicate significance at the 0.01 level, and ‘ns’ indicates non-significance.

During the heading stage of quinoa, the interaction among soil depth, nitrogen (N) level, and fertilizer type significantly affected soil SWC (*P* < 0.01). From 2022 to 2023, the SWC in the 0–1 m soil layer initially increased and then decreased with increasing soil depth, typically peaking around the 60 cm soil layer. In 2022, there were no significant differences among the fertilizer treatments across the three N application levels, and the trend remained consistent for all treatments (Fig. 3a). In 2023, after the application of BM and SRF, the overall SWC in soil layers deeper than 60 cm remained between 16% and 18% (Fig. 4a).

#### Anthesis stage

During the anthesis period, the interaction between soil depth, N level, and fertilizer type in 2022 significantly influenced SWC (*P* ≤ 0.01) (Fig. 3b). As soil depth increased, soil SWC initially increased and then decreased. The SRF treatment demonstrated high water use efficiency. In 2023, the interaction of these three factors significantly impacted soil SWC (*P* ≤ 0.01) (Fig. 4b). As soil depth increased, soil SWC exhibited a decreasing trend. Furthermore, there were significant differences in soil SWC among the various fertilizer types, particularly at the N2 level.

#### Pre-grouting stage

Before grouting, the combined interaction of soil depth, N level, and fertilizer type significantly affected soil SWC (0.05 < *P* ≤ 0.01) (Fig. 3c and 4c). In 2022, soil SWC exhibited a decreasing trend as soil layers increased, with no significant differences in SWC among the three N levels. SWC remained stable at approximately 13% in soil layers exceeding 40 cm (Fig. 3c). In 2023, SWC showed a trend of initially increasing and then decreasing with increasing soil depth. Notably, SRF significantly enhanced the infiltration of SWC into deeper soil layers, maintaining SWC at 17% in soil layers below 60 cm for both the N2 and N3 application levels (Fig. 4c).

#### Post- grouting stage

After grouting, the interaction among soil depth, N level, and fertilizer type was highly significant (*P* ≤ 0.01) (Fig. 3d and 4d). The overall change in soil SWC across the two growing seasons was insignificant. Specifically, in 2022, the vertical distribution of soil SWC showed an initial increase followed by a decrease, remaining within the range of 11–14%. In 2023, the SWC maintained levels between 14% and 16%.

#### Maturity stage

During the maturity period in 2022, the interaction between soil depth, N level, and fertilizer type was significant (*P* < 0.01) (Fig. 3e). As soil layers increased, the soil SWC exhibited an upward trend. At the N1 level, the soil SWC of the NPK treatment was comparable to that of the control group (CK) across all soil layers. However, at the N2 and N3 levels, the soil SWC of the NPK treatment was significantly enhanced. Both BM and SRF reached their highest SWC at the N2 level, with no significant difference between them. Soil SWC below the 20 cm depth was maintained at 12–13%. In 2023, the interaction between soil depth, N level, and fertilizer type remained significant (*P* < 0.01) (Fig. 4e). The SWC of BM and SRF increased with rising soil layers. Compared to N1, both N2 and N3 N levels significantly elevated the soil SWC of BM and SRF, maintaining an overall range of 13–15%.

### Soil organic matter (SOM), alkali hydrolyzed N (AH-N), available P (AP) and available K (AK)

**Fig. 5:**
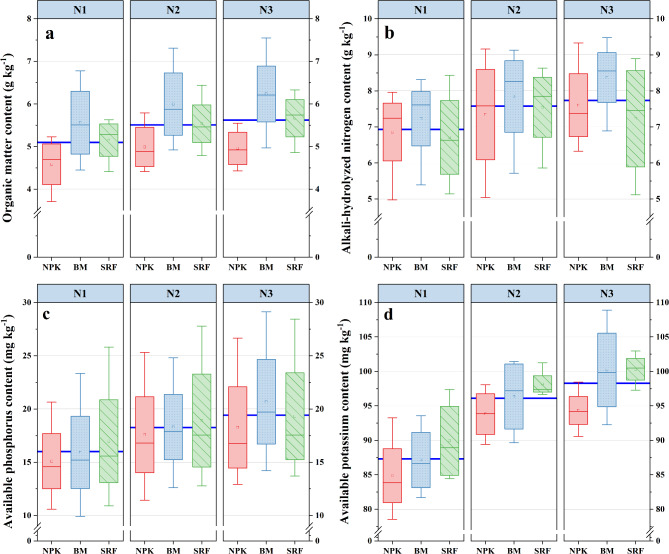
Effects of different nitrogen application levels and fertilizer types on soil organic matter (SOM-a), alkali-hydrolyzed N (AH-N-b), available P (AP-c), and available K (AK-d).

Fertilizer type and N level can significantly increase SOM content, and there are significant differences among different treatments at different stages (*P* ≤ 0.01) (Table S1). During the mature stage, the SOM content peaked, with increases ranging from 9.1 to 57.6% compared to the CK that did not receive any fertilization. Among the various fertilizer treatments, BM was particularly effective, significantly increasing the SOM content to its highest level at the N3 application level (Fig. 5).

The type of fertilizer, N level, and their interaction with the growth period all have a significant impact on AH-N, AP, and AK (*P* < 0.05) (Table S1). Both BM and SRF have a significant promoting effect on soil N, phosphorus, and potassium nutrients (Fig. 5). The accumulation rate of AH-N nutrients is highest from the heading stage to the anthesis, while the growth rate of AP and AK is highest from the anthesis to the filling stage. The content of AH-N, AP, and AK all peaks during the filling stage and decreases as the N application level increases up to N3. Among all treatments, the BM treatment had the highest content of AH-N, AP, and AK in the mature stage those were 27.7%, 50%, and 44.1% higher than CK, respectively. At the N2 level, there was no significant difference between the SRF and BM treatments. It was worth noting that the AK content in the surface soil was particularly high at the N3 N application level, with the BM treatment reaching its peak at this level. The BM treatment results in an increase in AK content by 5.7–11% compared to other fertilizers.

#### Soil urease (S-UE) and sucrase activity (S-SC)

**Fig. 6:**
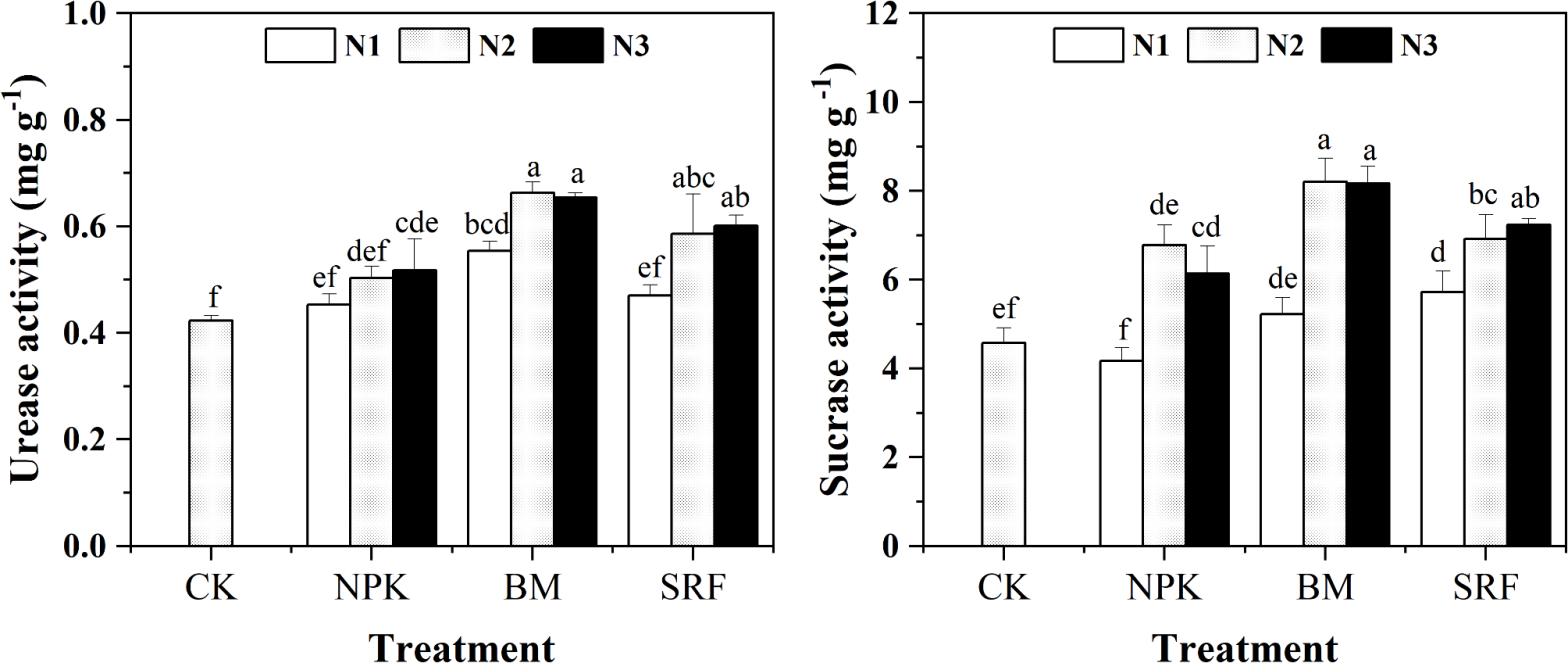
Effects of different nitrogen application levels and fertilizer types on Soil Urease (S-UE), Sucrase activity (S-SC) of quinoa. The error bars for each treatment represent the standard error of the mean (*n* = 3). Different letters on the bars indicate statistical significance following Duncan’s multiple range test (*P* < 0.05). Different lowercase letters indicate significant differences between different treatments (*P* < 0.05).

Results reveled that fertilizer types and N application levels can significantly increase S-UE and S-SC activities (Fig. 6). Soil S-UE and S-SC activities of the BM treatment were significantly higher than those of CK and other fertilization treatments, the S-UE activity of the BM treatment was 28.3% and 10.3% higher than that of the NPK and SRF, respectively. As the N application level increased, the S-SC activity of the NPK and BM exhibited a trend of first increasing and then decreasing. When compared to CK, each fertilizer type increased S-SC activity by 48.3%, 79.5%, and 58.3% respectively. Therefore, the level of N application had little effect on the activity of S-UE and S-SC; the primary influence may be attributed to the selection of fertilizer types.

### Plant height, leaf area (LA), above-ground biomass and yield components

N level and the type of fertilizer have a significant influence on the plant height of mature quinoa (*P* < 0.05), which first increases and then decreases with the increase of N application level. The highest effect is achieved in the SRF treatment, which is 16.7% higher than the CK (Table 2).

The LA and above-ground biomass of mature quinoa increase with the increase of N application level, and the effects of N application level, fertilizer type, and their interactions are significant (*P* < 0.05). The response of LA to the BM treatment was the most pronounced, achieving the highest level at N3, which was 4.5–15.1% greater than that observed under other fertilization treatments. Above-ground biomass of mature quinoa initially increased and then decreased as N application levels rose, with the except of the NPK treatment, which continued to increase with higher N levels. Compared to the CK treatment, NPK, BM, and SRF increased above-ground biomass by 83.4%, 69.1%, and 101.9%, respectively (Table 2). N level, fertilizer type, and their interaction have a highly significant effect on the branch number and main spike length of quinoa (*P* ≤ 0.01). All treatments significantly increased the branch number and main ear length of quinoa. At the N2 level, the branch number reached its highest level for all three fertilizer types. Similarly, at the N2 level, the main spike length of the BM and SRF treatments peaked.

**Table 2:** Mature plant height, leaf area, above-ground biomass, and yield composition of quinoa in two growing seasons under different fertilizer types and nitrogen levels.

Treatment		Plant height (cm)	Leaf area (cm^2^)	Above-ground biomass (g plant^− 1^)	Number of branches	Main spike length (cm)
Nitrogen level	Fertilizer type					
N0	CK	184.75 e	18.72 e	86.36 f	15.66 g	29.49 h
N1 (90 kg ha^− 1^)	NPK	188.46 e	19.62 de	103.91 e	17.24 f	34.93 g
	BM	190.96 de	20.06 d	95.59 ef	20.58 d	36.64 fg
	SRF	199.30 cd	21.50 c	126.33 d	19.69 e	37.76 ef
N2 (120 kg ha^− 1^)	NPK	201.78 bc	21.78 c	122.08 d	21.40 c	39.45 e
	BM	210.95 ab	24.12 b	146.07 bc	23.28 b	46.46 b
	SRF	215.65 a	24.51 ab	174.38 a	24.52 a	50.78 a
N3 (150 kg ha^− 1^)	NPK	205.99 bc	22.26 c	158.37 ab	20.04 de	42.61 d
	BM	205.55 bc	25.62 a	136.71 cd	22.71 b	44.00 cd
	SRF	209.87 ab	24.24 b	156.26 b	22.81 b	45.45 bc
Nitrogen level (N)		**	**	**	**	**
Fertilizer type ( F)		*	**	**	**	**
N×F		ns	*	*	**	*

NPK, BM, and SRF denote three types of fertilizers, compound fertilizer, bio-bacterial fertilizer, slow-release fertilizer, respectively. CK is the control without fertilization. Different letters (a–h) in the same column indicate statistical significance following Duncan’s multiple range test at (*p* ≤ 0.05). N、F and N × F respectively represent nitrogen level, fertilizer type, and their interaction, * indicating a significant effect (*P* < 0.05), and ** Indications a highly significant effect (*P* < 0.01), “ns” represents no significant difference.

#### Grain yield and water use efficiency (WUE)

N level, fertilizer type, and their interaction have a significant impact on the yield of quinoa (*P* ≤ 0.01) (Table 3). NPK treatment showed an increase with the rise in N application level, and increased by 65.2% and 68.8% respectively, compared to CK, for two consecutive years. BM and SRF treatments displayed a trend of initially increasing and then decreasing with the increase in N application level. For two consecutive years, SRF treatment yielded the highest in N2, outperforming both NPK and BM treatments by 15.6% and 10.5% (2022), 11.2% and 8.8% (2023). Compared to the N2 and N3 levels, the yield of quinoa at the N1 level decreased by 23–38.4% in 2022 and by 29.9–45.3% in 2023.

N level, fertilizer type, and their interaction exhibit a significant influence on WUE (*P* ≤ 0.01) (Table 3). In 2023 WUE of each fertilizer increased by an average of 18.4% (NPK), 16% (BM), and 14.2% (SRF) compared to previous year. Notably, SRF demonstrated a substantial boost in WUE, achieving increases of 16.2% and 10.8% in 2022, and 12.1% and 9.2% in 2023, relative to NPK and BM treatments, respectively. Furthermore, the WUE at N2 and N3 N levels surpassed that at the N1 level.

**Table 3:** Grain yield and water use efficiency(WUE) of quinoa in two growth seasons under different fertilizer types and nitrogen fertilizer levels.

Treatment		Grain yield (kg ha^− 1^) GA		Water use efficiency WUE	
Nitrogen level	Fertilizer type	2022	2023	2022	2023
N0	CK	2224.41 e	2559.79 g	3.09 f	3.62 f
N1 (90 kg ha^− 1^)	NPK	2808.75 d	2974.15 f	3.94 e	4.25 e
	BM	3124.41 c	3398.91 e	4.41 d	4.89 d
	SRF	3068.75 c	3529.21 de	4.37 d	5.10 d
N2 (120 kg ha^− 1^)	NPK	3158.75 c	3645.39 d	4.43 d	5.23 d
	BM	3842.16 b	4415.98 b	5.44 b	6.31 b
	SRF	4247.32 a	4804.25 a	6.03 a	6.89 a
N3 (150 kg ha^− 1^)	NPK	3674.41 b	4320.06 b	5.19 c	6.15 bc
	BM	3766.64 b	4056.49 c	5.40 bc	5.83 c
	SRF	3842.16 b	4409.98 b	5.48 b	6.29 b
Nitrogen level (N)		**	**	**	**
Fertilizer type ( F)		**	**	**	**
N×F		**	**	**	**

Different letters in the same column indicate statistical significance following Duncan’s multiple range test at (*p* ≤ 0.05). N、F and N × F respectively represent nitrogen level, fertilizer type, and their interaction, * indicating a significant effect (*P* < 0.05), and ** Indications a highly significant effect (*P* < 0.01).

#### Economic benefit of quinoa

Different fertilizer types and N levels exhibited significant variations in the economic benefits of quinoa production (Table 4). In the absence of fertilization, after deducting other costs and a seed cost of 3,500 yuan (CNY) per hectare, the economic benefit per hectare amounted to 35,514.6 CNY. When considering the costs of fertilizer, seeds, and other expenses, BM and SRF were found to be most economical at the N2 N level, whereas NPK was most economical at the N3 level. Fertilization significantly enhanced economic benefits, SRF yielded economic benefits that were 7,434 CNY and 9,040 CNY higher than those of NPK and BM, respectively. Overall, the highest economic benefits for SRF were observed at the N2 N level.

**Table 4:** Economic benefit of quinoa production (per hectare) under different fertilizer types and nitrogen level.

Treatment		Cost (CNY per ha^− 1^)			Yield	Gross gain (CNY)	Economic benefit
		Fertilizer cost	Other cost	Total cost (CNY)			
N0	CK	0	5000	5000	2392.10	44014.6	39014.6
N1 (90 kg ha^− 1^)	NPK	648	5000	5648	2891.45	53202.7	47554.7
	BM	1425	5000	6425	3261.66	60014.5	53589.8
	SRF	1324	5000	6324	3298.98	60701.2	54377.7
N2 (120 kg ha^− 1^)	NPK	864	5000	5864	3402.07	62598.1	56734.1
	BM	1900	5000	6900	4129.07	75974.8	69075.2
	SRF	1765	5000	6765	4525.79	83274.4	76509.4
N3 (150 kg ha^− 1^)	NPK	1080	5000	6080	3997.24	73549.1	67469.1
	BM	2375	5000	7375	3911.57	71972.8	64598.2
	SRF	2206	5000	7206	4126.07	75919.6	68713.8

Other costs include pesticides, machinery fuel, labour cost, and other costs. CNY: Chinese yuan. The yield and total cost are averaged across two years.

#### Principle component analysis (PCA) of soil moisture and nutrients

**Fig. 7:**
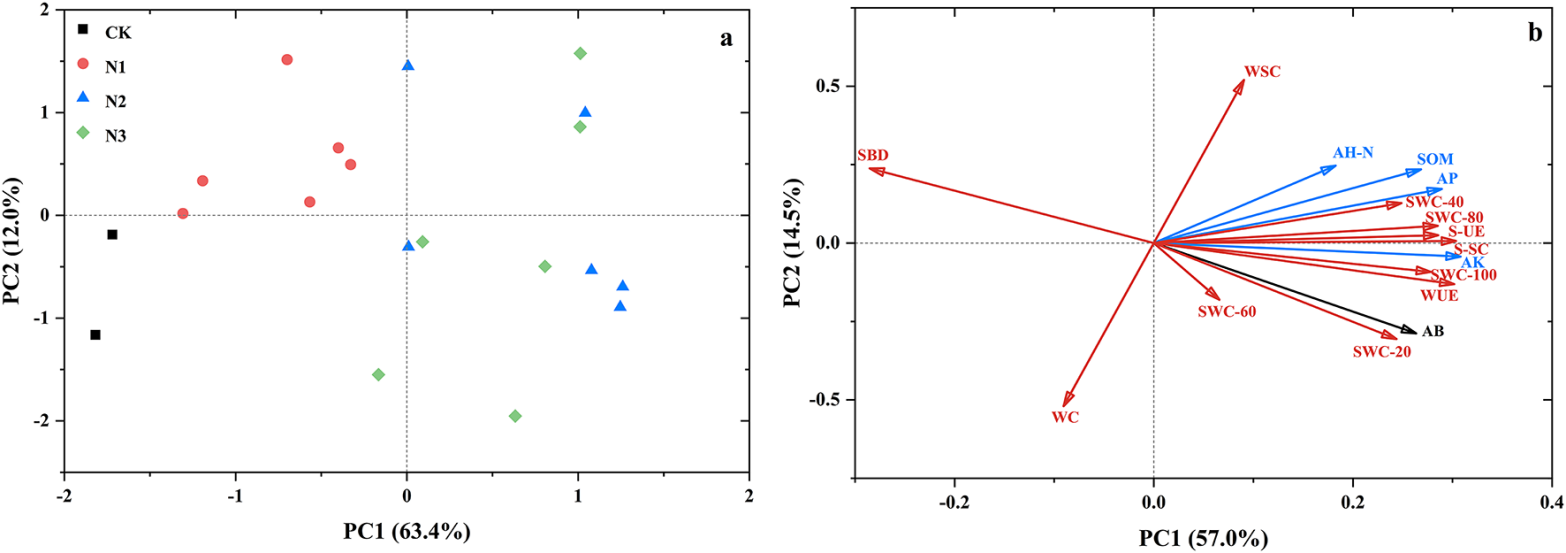
PCA analysis of soil nutrient and water related indicators under different nitrogen application levels and fertilizer types. S-UE, Soil urease; S-SC, Soil sucrase; SOM, Organic matter; AH-N, Alkaline hydrolysis nitrogen; AP, Available phosphorus; AK, Available potassium; MC-20, 0–20 cm moisture content; MC-40, 20–40 cm moisture content; MC-60, 40–60 cm moisture content; MC-80, 60–80 cm moisture content, MC-100, 100 cm moisture content; WSC, Mature water storage capacity; WC, Water consumption; WUE, Water use efficiency.

**Fig. 8:**
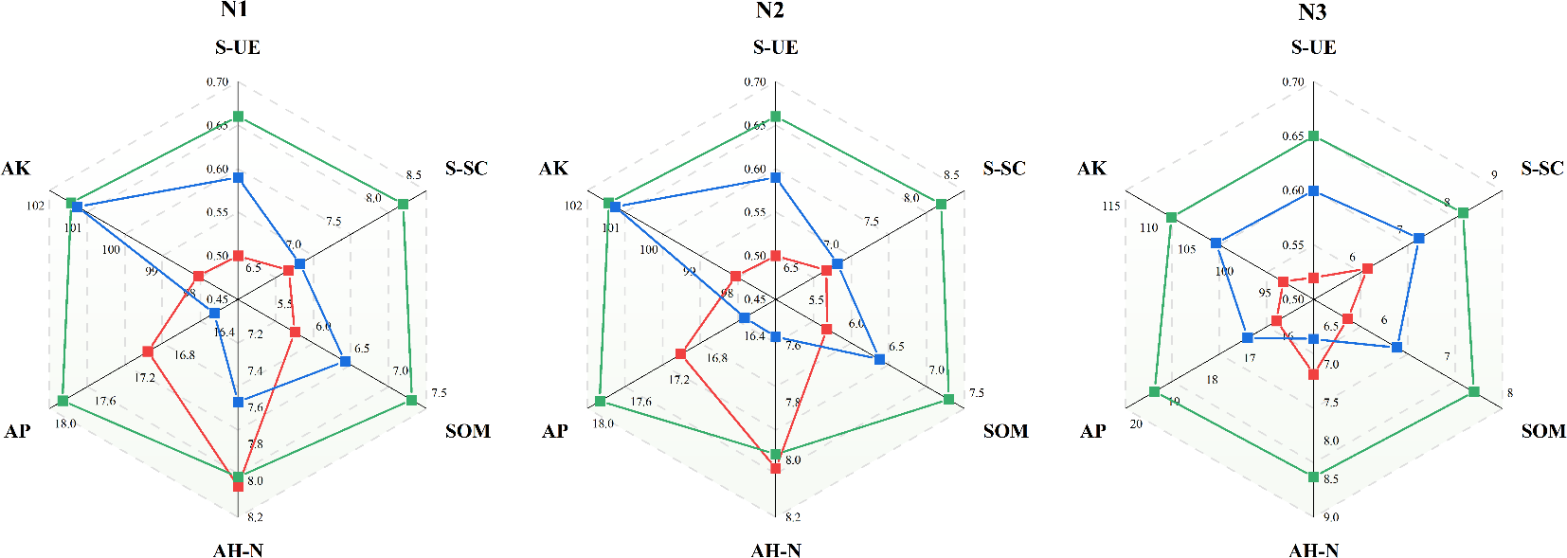
Effects of different nitrogen application levels and fertilizer types on soil nutrients.

The effects of varying nitrogen application levels on soil moisture and nutrient content exhibited distinct differences. Upon conducting dimensionality reduction analysis (Fig. 7a), it was observed that there were minimal differences between N1 and CK, whereas N2 and N3 exhibited similar trends. The disparities among the treatments were statistically significant. Regarding individual indices (Fig. 7b), soil bulk density and water consumption displayed negative correlations with other indicators. Soil water storage showed a negative correlation with the moisture content of the 60 cm soil layer, whereas other indicators demonstrated significant positive correlations. All types of fertilizers significantly enhanced soil nutrient levels (Fig. 8). However, the effects varied among different fertilizer treatments. Specifically, the BM treatment had a more pronounced effect on overall soil nutrient levels, while SRF notably increased soil urease activity and available potassium content. Additionally, NPK fertilizer significantly increased the soil alkali-hydrolyzed nitrogen content.

#### Path analysis of soil moisture and nutrients and yield

To clarify the direct effects of different fertilizer types and nitrogen application levels on quinoa yield, path analysis was conducted for each index and yield (Table 5). According to the correlation coefficients, soil available potassium and sucrase activity had the highest correlation with yield, reaching 0.888 and 0.846, respectively. The highest direct path coefficient was for available potassium, indicating its importance as an index affecting quinoa yield. Additionally, the indirect path coefficient showed that available phosphorus can significantly increase quinoa yield through the action of available potassium.

**Table 5:** Path analysis of soil indicators on yield of quinoa under different fertilizer types and nitrogen fertilizer levels.

Character		Correlation coefficient	Direct diameter coefficient	Indirect path coefficient				
				X_1_-Y	X_2_-Y	X_3_-Y	X_4_-Y	X_5_-Y
Soil enzyme	Urease(X_1_)	0.763	0.161		0.137			
	Sucrase(X_2_)	0.846	0.709	0.601				
Soil chemical property	Organic matter(X_1_)	0.737	0.412		0.229	0.259	0.253	
	Alkali hydrolyzed nitrogen(X_2_)	0.426	0.277	0.154		0.150	0.166	
	Available phosphorus(X_3_)	0.718	0.09	0.057	0.049		0.072	
	Available potassium(X_4_)	0.888	0.873	0.535	0.523	0.699		
Moisture content of each soil layer	0–20 cm(X_1_)	0.506	0.313		0.308	0.021	0.137	0.146
	20–40 cm(X_2_)	0.548	0.274	0.270		0.009	0.138	0.135
	40–60 cm(X_3_)	0.548	0.087	0.006	0.003		0.048	0.057
	60–80 cm(X_4_)	0.838	0.573	0.252	0.288	0.319		0.388
	80–100 cm(X_5_)	0.806	0.463	0.216	0.228	0.304	0.314	

## Discussion

Soil movement cannot occur without precipitation and wind. Precipitation is the most direct driving force behind hydrological processes, primarily taking the form of liquid water^30^. However, in high-altitude regions, the ratio of solid to liquid precipitation depends on the characteristics of the low-pressure system, making precipitation an even more powerful direct driving force^31^. Overall, soil water content (SWC) within the 0–100 cm depth range in 2023 was observed to be higher than in 2022, primarily due to the significantly greater total rainfall recorded in 2023 compared to 2022 (Fig. 1). However, from July to September 2023, temperatures rose significantly and persisted for an extended period. These temperature fluctuations negatively impacted soil moisture content and soil dynamics. Conversely, these temperature changes had a positive effect on groundwater flow in high-altitude areas^32^. Therefore, throughout the entire grouting period, even during historically high temperatures, the SWC in 2023 remains significantly higher than that observed in 2022. Quinoa exhibits a slow growth rate before heading, and its limited surface area, combined with temperature fluctuations due to rainfall, can lead to reduced transpiration rates. Notably, the plants have minimal water requirements during this period. However, as they transition to the flowering stage, their water consumption gradually increases, signaling the start of effective growth^10^. As a result, the availability of water during the flowering and filling stages has a particularly pronounced impact on quinoa yield^33^. In 2022, observations from the flowering stage onward indicated a gradual increase in SWC in plots treated with bio-microbial fertilizers (BM). There was no significant difference in SWC between the BM and slow-release (SRF) treatments in soil layers below 60 cm, especially at the N2 and N3 levels (Fig. 3b-d). In 2023, precipitation experienced a notable surge during the flowering stage and the initial grain-filling stage, persisting over an extended period (Fig. 1). This led to the highest SWC observed throughout the entire growth cycle, adequately meeting the water requirements of quinoa from flowering to grain-filling. The frequent precipitation supplied sufficient moisture to the soil, accelerating the hydrolysis of N fertilizers^34^, consequently, seed growth flourished in the absence of water stress, ultimately determining the potential yield^35^. In the vertical direction, during periods other than the flowering stage, the SWC of the surface soil exhibited a trend of initially increasing and then decreasing as the soil layer deepened. Notably, the SWC of the surface soil was the lowest, which could be attributed to water loss through surface soil evaporation^12^. Under the treatment of BM and SRF, significant improvement in surface soil structure and infiltration of rainwater from 0 to 1 m into deep soil layers, maintain the SWC of soil layer below 60 cm at 11–13% (2022) and 14–15% (2023). During the whole growth period, the SWC of soil layer below 60 cm treated by SRF increased by 3–16.2% (2022), and 4.6–25.2% (2023) compared with that treated by NPK treatment, improving WUE through effective fertilizer management strategies is an urgent and critically important need^36^. Under the three types of fertilizers, the WUE of N2 level BM and SRF treatment reached the highest level. Therefore, in this section, we confirm the hypothesis that fertilizer types can regulate the dynamic distribution of soil moisture. As the N application level increased, the potential of BM and SRF was inevitably weakened (Table 2). The WUE of the NPK treatment increased with the rise in N application level, potentially due to the synergistic effect of NPK treatment on water and N interactions.

The content of available nutrients in the soil primarily mirrors the dynamic equilibrium between soil mineralization and plant absorption processes^37^. The findings of this study reveal that, in comparison to other fertilizers, BM significantly elevates soil SOM content (Fig. 5a), this is attributed to the rich organic matter content of the BM employed in this experiment. These microorganisms, possessing growth capabilities, enter the soil and contribute to an increase in SOM content. Furthermore, BM significantly increased the content of AP at N2 and N3 levels, suggesting that the application of microbial fertilizer substantially improves soil organic matter status, mitigates phosphorus fixation, and consequently boosts soil phosphorus availability^38^, this confirms our hypothesis that specific fertilizer types have a crucial impact on soil nutrients, even when N application levels are held constant. This effect varies with the level of N application, at the N1 and N2 levels, soil AP and AK nutrients attained higher levels in the late growth stage when treated with SRF (Fig. 5c-d). This was primarily due to the differing release rate of SRF compared to ordinary fertilizers, which allows for gradual nutrient supply at various growth stages. This ensures the availability of soil nutrients and maintains soil enzyme activity in the middle and later stages of growth, thereby enhancing the soil’s nutrient supply capacity throughout the harvest period^39^.

Soil enzyme activity is crucial for soil nutrient cycling and organic matter decomposition^40^. The level of soil enzyme activity serves as an indicator of the scale of soil mineralization and the intensity of soil productivity, ultimately influencing the effectiveness of soil nutrient utilization, a significant positive correlation exists between soil fertility and soil enzyme activity, generally, higher nutrient content in the soil tends to result in elevated levels of soil enzyme activity^41,42^. In this study, the application of microbial fertilizers resulted in a significant increase in soil nutrient levels and enhanced the activities of S-UE and S-SC. Conversely, the use of conventional compound fertilizers led to a reduction in soil enzyme activity (Fig. 6). These findings align with previous research, which reported that microbial fertilizers significantly elevate soil S-UE and S-SC activity, as well as increase the content of SOM, AP and AK^43^, organic matrices, abundant in nutrients, positively influence N fixation, phosphorus solubilization, potassium release, and other processes^44^. Furthermore, microbial fertilizers greatly facilitate the absorption and utilization of AH-N and AK by plants and other soil microorganisms^45^. Notably, the activity of S-UE diminishes with increasing fertilizer application, potentially due to the presence of both amino-N fertilizers and urea as N sources in the fertilizer^46^. N fertilizers contain NH_4_^+^, and the enzymatic reaction products also include NH_4_^+^, this suggests that repeated applications of NH_4_^+^ may inhibit the microbial induction of S-UE^47^. Previous studies have demonstrated that, despite this potential inhibition, the addition of N fertilizers can significantly elevate soil S-UE and S-SC activity^48–50^. The results of this experiment demonstrate consistency, with three N application levels resulting in varying degrees of increase in soil enzyme activity. Specifically, at the N2 level, the soil S-UE and S-SC activities of the BM reached their peak. Conversely, the S-UE activities of both the NPK and SRF peaked at the N3 level, although these values were not significantly different from those at the N2 level. Notably, under BM treatment, soil S-UE and S-SC exhibited the highest activities, potentially attributed to the enhancement of microbial activity. Soil properties exert both direct and indirect influences on S-UE activity, with soil nutrients at the mature stage exhibiting a particularly significant impact on its activity levels. Appropriate nutrient release fosters root growth, optimizes the soil microbial environment, stimulates the secretion of additional enzymes and enzymatic substances, and thereby significantly enhances enzyme activity^51^.

There is a significant bidirectional effect of fertilizer type and N level on the dry matter quality, yield, and composition of quinoa (Tables 2 and 3) (*P* ≤ 0.01). Papastylianou^52^ reported that fertilizer type had significant effects on quinoa traits. Al-Naggar^53^ showed that fertilizer type, N application amount and year had significant effects on 25 out of the 28 traits studied. When the N application rate increases from 0 to 120 kg ha^− 1^, the yield increases from 1.79 t ha^− 1^ to 3.59t ha^− 1^^52^. The grain yield of quinoa with 120 kg ha^− 1^ N was increased by 94%^54^. This experiment was consistent with the above results. When the N rate was 120 kg ha^− 1^, the increases in NPK, BM, and SRF compared to CK were 42%, 72.7%, and 90.9% respectively in 2022, and 42.4%, 72.5%, and 87.7% respectively in 2023. In 2022, the application of the three types of fertilizers at the N3 level had no significant effect on yield. However, the yield of quinoa treated with BM and SRF decreased in both growing seasons as the N application rate increased to the N3 level (Table 2). This phenomenon indicates that when the amount of fertilizer applied exceeds a certain threshold, the plant cannot continue to absorb for utilization^55^. The yield of quinoa treated with SRF was higher than that treated with BM and NPK under the same N application. In terms of fertilizer type, SRF significantly promoted the growth and yield of quinoa, and consistent results were also found on other crops. Tomato^56^, corn^57^, wheat^58^, etc. all achieved the highest yield under SRF treatment, which may be related to the ability of slow-release N fertilizer to meet the N demand in the middle and late stages of plant growth^59^.

The above-ground dry matter quality is an important index affecting crop yield, and the dry matter accumulation of quinoa treated with SRF reached the highest level at all N application rates. This was primarily due to the fact that SRF maintains a relatively stable nutrient supply throughout the growing season and continues to release nutrients in the later stages of crop growth, thereby enhancing the dry matter accumulation rate^60,61^. The increase in above-ground biomass was primarily attributed to the enhanced growth rate of crops, coupled with better accumulation in terms of plant height, branch number, and main spike length, all of which contributed to higher above-ground biomass. Studies have demonstrated that SRF treatment results in higher dry matter weight, ear length, and grain yield compared to urea treatment^62^. In this experiment, compared to BM and NPK treatments, SRF significantly increased the plant height of quinoa (2.2%, 4.7%), branch number (5.3%, 22.3%), main spike length (9.3%, 19.2%) (Table 2). Leaf area, as an important agronomic trait, effectively encapsulates the complexity arising from the interplay of internal and external factors that influence plant canopy structure^63^. In BM treatment quinoa leaf area was observed to increase by 4.5% and 15.1% over SRF and NPK treatments, respectively. This increase in leaf area leads to greater light interception, thereby enhancing the photosynthetic rate and light use efficiency, which ultimately contributes to increased crop yield^64^.

In our hypothesis, we assumed that fertilizer type could regulate the dynamic distribution of soil water and enhance rainfall infiltration into deep soil layers. Fertilizer type played a significant role in soil moisture, nutrient levels, and crop yield when N levels were consistent. The results showed that at a level of 120 kg ha^− 1^, BM treatment significantly increased soil nutrients and enzyme activity. Moreover, the water content, yield, and composition of the 60–100 cm soil layer under SRF treatment were notably higher than those observed under other fertilization methods. Consequently, selecting the appropriate fertilizer type can enhance soil water use efficiency. Additionally, we found that each production index exhibited a strong response to yield in the 80–100 cm soil layer at maturity, but had no significant correlation with the intermediate 40–60 cm soil layer. Therefore, the research results suggest that fertilizer selection can be a priority strategy for improving water use efficiency and yield in high-altitude areas where irrigation is not feasible. This finding is not limited to this experimental site, but has broader implications for enhancing yield and water productivity in quinoa planting regions across China and its neighboring high-altitude countries.

## Conclusion

In this study, we examined the vertical and seasonal variations in quinoa growth, soil nutrients, moisture dynamics, enzyme activity, and yield in relation to different fertilizer types and nitrogen (N) application levels. Our findings indicated that slow-release and bio-microbial fertilizers significantly enhanced quinoa yield and water use efficiency, while also improving soil conditions and increasing the accumulation of soil moisture, nutrients, and enzymes. Specifically, at an N application rate of 150 kg ha^− 1^, bio-microbial fertilizer optimized soil alkali-hydrolyzed N, available phosphorus, available potassium, as well as sucrase and urease activities; however, the differences were not significant when compared to the 120 kg ha^− 1^ level. Additionally, the slow-release fertilizer promoted crop growth at 120 kg ha^− 1^, reduced soil bulk density, regulated rainfall infiltration, and ultimately improved food yield and water efficiency. The compound fertilizer was most effective at an application rate of 150 kg ha^− 1^, while slow-release and bio-microbial fertilizers yielded the best results at 120 kg ha^− 1^. A comprehensive economic analysis revealed that applying 120 kg ha^− 1^ of slow-release fertilizer proved to be highly valuable, ensuring sustained increases in quinoa yield and water use efficiency.

## Electronic supplementary material

Below is the link to the electronic supplementary material.

Supplementary Material 1

## Data Availability

All data generated or analysed during this study are included in this published article.
