# Supplementary material for: Fertilizer types and nitrogen rates integrated strategy for achieving sustainable quinoa yield and dynamic soil nutrient-water distribution at high altitude
Source: Sci Rep. 2025 Feb 15;15:5599. doi: 10.1038/s41598-025-89572-2 (PMC11829954; doi:10.1038/s41598-025-89572-2)
Supplement: Supplementary file 1 — Supplementary Material 1 [file 41598_2025_89572_MOESM1_ESM.docx]

**Table S1. The analysis of variance indicates the effect of different N fertilizer rates and fertilizer types on organic matter (SOM), Alkali hydrolyzed nitrogen (AH-N), available phosphorus (AP) and available potassium (AK) content at different growth stages of quinoa.**

| Treatments |  | SOM | AH-N | AP | AK |
| --- | --- | --- | --- | --- | --- |
| Nitrogen rate  (N) | 90 | 5.10b | 6.93b | 16.00b | 87.32b |
|  | 120 | 5.51a | 7.58ab | 18.27a | 96.12ab |
|  | 150 | 5.62a | 7.74a | 19.42a | 98.28a |
| Fertilizer type  (F) | NPK | 4.84b | 7.27b | 16.98b | 91.01a |
|  | BM | 5.93a | 7.82a | 18.30a | 94.57a |
|  | SRF | 5.45ab | 7.16ab | 18.40a | 96.13a |
| Period (P) | Heading | 4.55c | 5.61c | 12.12c | 93.07ab |
|  | Anthesis | 5.22b | 7.80b | 17.19b | 90.33b |
|  | Grouting | 5.57b | 8.81a | 25.76a | 97.62a |
|  | Maturity | 6.29a | 7.43b | 16.52b | 94.60ab |
| Nitrogen level (N) | | ** | ** | ** | ** |
| Fertilizer type (F) | | ** | ** | ** | ** |
| Period (P) | | ** | ** | ** | ** |
| N×F | | ns | * | ** | ns |
| N×P | | ns | ** | * | ** |
| F×P | | ** | * | ** | ** |
| N×F×P | | ns | ** | * | * |

Note: * indicate significance at the 0.05 level, ** indicate significance at the 0.01 level, and “ns” indicates non-significance. Different letters following mean (n=3) indicate significant differences at p<0.05 using the Duncan’s multiple range test.
